# CNS-associated macrophages shape the inflammatory response in a mouse model of Parkinson’s disease

**DOI:** 10.1038/s41467-023-39061-9

**Published:** 2023-06-26

**Authors:** Maximilian Frosch, Lukas Amann, Marco Prinz

**Affiliations:** 1 Faculty of Medicine, Institute of Neuropathology, University of Freiburg, Freiburg, Germany; 2 Signalling Research Centres BIOSS and CIBSS, University of Freiburg, Freiburg, Germany

**Keywords:** Glial biology, Parkinson's disease

## Abstract

In an alpha-synuclein (α-syn) model of Parkinson’s disease (PD), Schonhoff and colleagues have shown that central nervous system (CNS)-associated macrophages (CAMs), but not microglia, potentially orchestrate CD4^+^ T cell recruitment and mediate an α-syn-induced inflammatory makeup.

CNS tissue macrophages are typically subdivided into two groups: microglia reside within the CNS parenchyma, whereas CAMs are found at CNS interfaces, including the leptomeninges, perivascular space, and choroid plexus^1^. Microglia are relatively well characterized, and a plethora of physiological functions are known, including synaptic pruning, regulation of neuronal activity, and coordination of oligodendrocyte and myelin homeostasis^2^. In contrast, the role of CAMs in health and disease is much less understood. Only recently, researchers started elucidating the physiological role of these macrophage populations. Given their anatomical localization and continuous exposure to the cerebrospinal fluid (CSF), it seems self-evident that perivascular macrophages (pvMΦ) or leptomeningeal macrophages (mMΦ) monitor and clear the CSF from unwanted molecules and metabolites. In line with this notion, intravenously injected horseradish peroxidase that diffuses into the CSF, as well as intraventricular injected ferritin, has been shown to accumulate in CAMs^3,4^. Moreover, another study recently showed that CAMs are able to regulate the CSF flow by remodeling the arterial motion and are thus required for a proper and functional CSF flow^5^. In addition to the recent progress in illuminating the role of CAMs in steady state, several studies suggested an involvement and detrimental influence of CAMs in diverse pathologies. Their residence at CNS interfaces implies a role in recruiting peripheral immune cells to the CNS and exchanging molecules like cytokines or chemokines. For instance, the recruitment of autoreactive T cells into the CNS parenchyma in experimental autoimmune encephalomyelitis, a mouse model of multiple sclerosis, is a crucial step in disease initiation and highly dependent on antigen-presenting cells (APCs). A couple of studies revealed conventional dendritic cells, but not CAMs, as essential cell type for antigen presentation to primed T cells at CNS interfaces^6–8^. Nevertheless, upon disease induction, CAMs strongly expand, overt a context-dependent activated gene signature, and secrete pro-inflammatory cytokines and chemokines^6^. The establishment of such a pro-inflammatory milieu enhancing the recruitment of monocyte-derived macrophages or the expansion of T cells strongly suggests a detrimental role of CAMs during the disease course. However, not only during neuroinflammation, but also in neurodegenerative diseases such as Alzheimer’s disease (AD), a detrimental role of CAMs has been reported. In mouse models of AD, the selective depletion of CAMs leads to a reduction in vascular amyloid beta deposition^9^ as well as to an abrogated reactive oxygen production and cerebrovascular dysfunction^10^. Moreover, the authors identified CD36 and Nox2 as molecules that, upon deletion from CAMs, abolish the deleterious vascular effects of amyloid beta^10^. Another study showed that the inactivation of a single SR-BI allele is sufficient to impair pvMΦ response to amyloid beta and to enhance fibrillar amyloid deposition, again implicating a considerable influence of CAMs on disease course^11^. But also, a role of CAMs in chronic hypertension^12^, stroke^13^, or infections^14^ has been described.

Now, Schonhoff et al. add another layer of evidence that CAMs are active modulators of CNS pathologies. In their study^15^ published in *Nature Communications*, they took advantage of a rodent PD model based on an rAAV to induce α-syn overexpression in dopaminergic neurons of the substantia nigra pars compacta (SNpc). Subsequently, 6 months after viral injection, aggregation of phosphorylated α-syn and a loss of 25–30% of tyrosine hydroxylase (TH)-positive neurons can be observed in the SNpc. Moreover, this PD model exhibits an infiltration of CD4^+^ T cells into the midbrain that is highly dependent on MHC class II (MHCII) expression^16^.

## Identification of the pathophysiological relevant antigen-presenting cell type

**Fig. 1:**
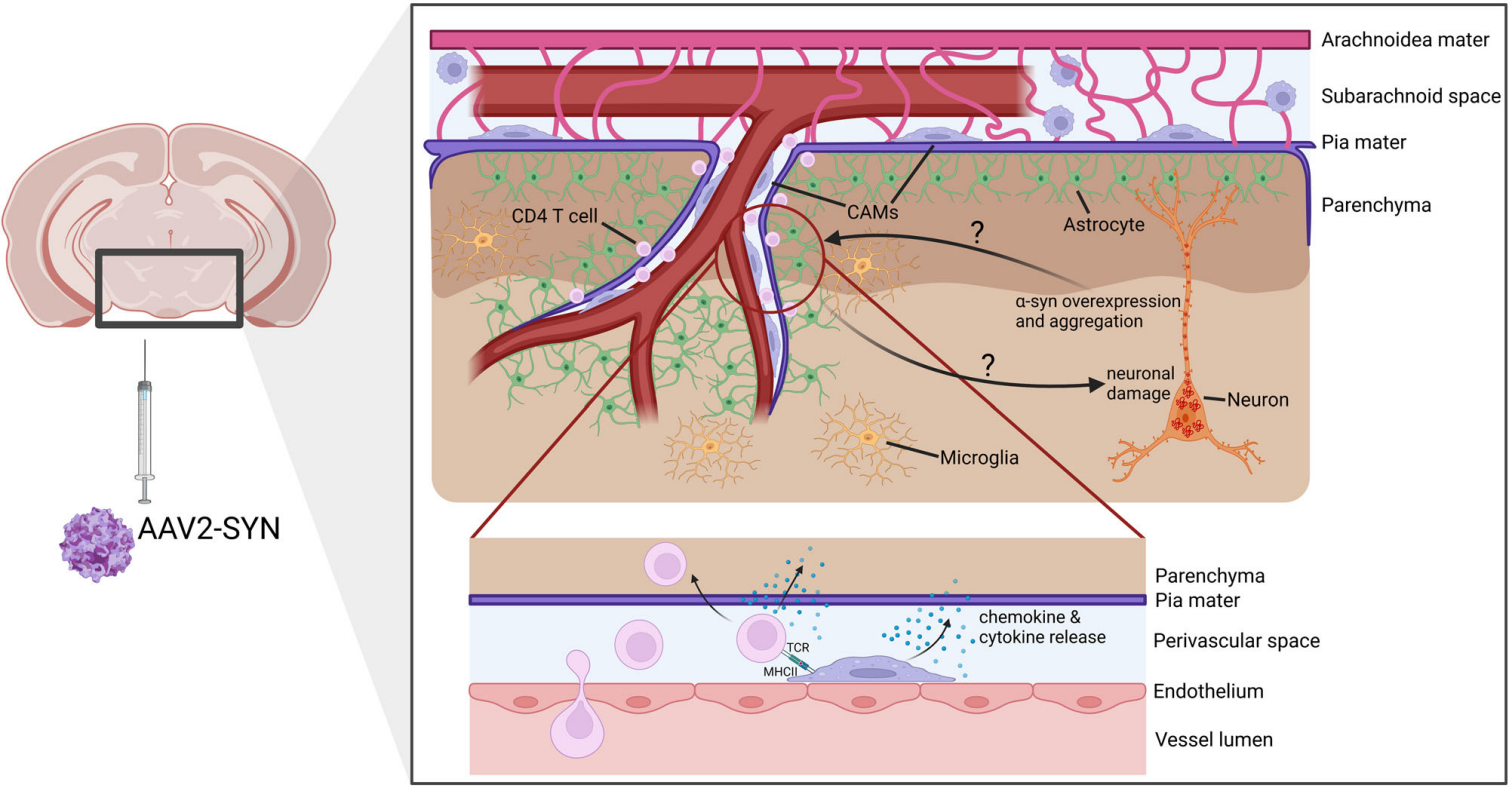
Potential mechanism of CAM involvement in PD. In a rodent PD model based on an rAAV- to induce α-syn overexpression, CD4+ T cells enter the perivascular space and, ultimately, the brain parenchyma. Schonhoff et al. could show that this process highly depends on the presence of CAMs, most likely due to antigen presentation via MHCII. However, it is still unclear how CAMs exactly are affected by α-syn overexpression (direct vs. indirect effects) and if the recruitment of CD4+ T cells affects the progression of neuronal damage (these open questions are indicated by arrows with question marks). Figure made using BioRender.

Since the pathophysiological relevant APC type was unidentified so far, the authors made use of the *Cx3cr1*^*CreERT2/+*^ *IAB*^*fl/fl*^ mouse line to delete MHCII from long-living brain myeloid cells. After tamoxifen induction, mice were transduced with the rAAV to induce α-syn overexpression. Even though the α-syn pathology was unchanged 4 weeks post-transduction, microglial activation and CD4^+^ T cell infiltration were reduced. Surprisingly, MHCII deletion in *Cx3cr1*^*CreERT2/+*^ *IAB*^*fl/fl*^ mice resulted in a neuroprotective effect 6 months post-transduction, evidenced by the absence of a significant loss of TH^+^ neurons in the SNpc. This experiment clearly points toward that antigen presentation by long-living brain myeloid cells, including microglia and CAMs, is indispensable for recruiting CD4^+^ T cells and, ultimately, neurodegeneration (Fig. 1).

In a second step, the authors aimed to characterize these CX3CR1^+^ cells during α-syn overexpression by performing single-cell RNA sequencing (scRNA-seq) to identify cell type-specific transcriptional changes. Indeed, two distinct major populations were identified and annotated as microglia and CAMs based on the expression of classical microglial (including *Sall1*, *Tmem119*, *Fcrls*) and CAM markers (such as *Mrc1*, *Clec12a*, *Ms4a7*). Further analysis revealed a clear expansion of microglia clusters displaying activated gene signatures (e.g., *Cst7*, *Lpl*, *Apoe*) in diseased mice. The genes defining these disease-associated clusters showed enrichment for pathways involved in phagocytosis, cytokine/chemokine signaling, and antigen presentation and processing. Taken together, scRNA-seq of CX3CR1^+^ cells uncovered substantial changes in the microglial transcriptional program and function.

This aberrant microglial gene signature raised the question whether microglial antigen presentation is required to initiate α-syn-induced CD4^+^ T cell recruitment. To test this hypothesis, the authors next specifically deleted MHCII from microglia using the Tmem119^CreERT2/+^ IAB^fl/fl^ mouse line. After induction of α-syn overexpression, lack of MHCII expression on microglia was confirmed by flow cytometry, whereas CAMs were still able to upregulate MHCII. Notably, no changes in the infiltration of peripheral immune cells (CD4^+^ and CD8^+^ T cells, monocytes) were observed in the Tmem119^CreERT2/+^ IAB^fl/fl^ mouse line. This clearly indicates that not microglia, but CAMs are the pathophysiological relevant cell type for T cell recruitment via antigen presentation.

## Evidence for disease-modulating role of CNS-associated macrophages

Having identified CAMs as the key players for leukocyte recruitment, the authors focused on the gene signatures of CAMs in the scRNA-seq dataset. Subsetting and re-clustering revealed eight sub-clusters of CAMs. Two clusters were massively expanded in the disease context and hence, the authors referred to these cells as “disease-activated” CAMs. The cluster-defining genes comprised *Cd68*, *Il1b*, *Ccl5*, *Cxcl10*, *Mmp14*, or *Gpnmb* among others. A pathway analysis further linked these disease-activated clusters to inflammatory cellular processes such as cytokine signaling or antigen processing and presentation, again suggesting a key role of CAMs in disease initiation and progression.

However, though the authors showed tremendous indirect evidence for a disease-modulating role of CAMs, a direct investigation of CAMs was still missing. To address this issue, they used clodronate liposomes to deplete CAMs specifically. Flow cytometry and immunohistochemistry confirmed a sufficient depletion of CAMs, but not microglia. Here, a dramatic reduction of MHCII expression in the SNpc was observed in the clodronate-treated animals, accompanied by a significant decrease in infiltrating CD4^+^ T cells and monocytes. In general, the depletion of CAMs highly recapitulated the phenotype seen in *Cx3cr1*^*CreERT2/+*^ *IAB*^*fl/fl*^ mice, confirming the requirement of CAMs for the recruitment of peripheral immune cells in the context of α-syn overexpression-induced pathology.

## Translation into the human disease condition: CNS-associated macrophages reside next to T cells in Parkinson’s disease brains

In a final step, Schonhoff et al. aimed to translate their findings into the human disease condition. Since they noted T cells and CAMs often in close proximity in the PD mouse model, they sought to identify such interactions in midbrain sections of human PD patients. Indeed, CAMs (identified as CD68^+^ perivascular cells) are significantly more often localized close to CD3^+^ T cells in PD brains than in control brains. Moreover, these T cells comprised both subsets, CD4^+^ and CD8^+^ T cells.

## Concluding remarks

In sum, Schonhoff et al.^15^ report that not microglia, but rather CAMs are the primary APCs initiating CD4+ T cell recruitment and an α-syn-induced inflammatory makeup in a mouse model of PD, to the best of our knowledge, a novel and potentially paradigm-shifting finding. For the first time, a study implicates a tremendous influence of CAMs in the disease initiation and progression in PD. These observations raise plenty of new questions in the field: How does α-syn overexpression alter CAMs? What exact mechanisms lead to T cell recruitment mediated by CAMs? CAM-specific Cre-lines^17^ can further help to delete MHCII molecules from these cells to gain more functional insights. In addition, are these findings of therapeutic usage? Do T cells recruited by CAMs contribute to neurodegeneration? Can a long-term depletion of CAMs prevent neuronal loss? And if so, does it change the behavioral outcome of diseased mice? Answering these questions will inform us about the nature and biology of CAMs and their relevance as therapeutic targets in PD.
